# Evaluation of Mesenchymal Stem Cell Therapy for Sepsis: A Randomized Controlled Porcine Study

**DOI:** 10.3389/fimmu.2020.00126

**Published:** 2020-02-07

**Authors:** Jan Horak, Lukas Nalos, Vendula Martinkova, Vaclav Tegl, Lucie Vistejnova, Jitka Kuncova, Michaela Kohoutova, Dagmar Jarkovska, Martina Dolejsova, Jan Benes, Milan Stengl, Martin Matejovic

**Affiliations:** ^1^First Medical Department, Faculty of Medicine in Pilsen, Charles University, Pilsen, Czechia; ^2^Faculty of Medicine in Pilsen, Biomedical Center, Charles University, Pilsen, Czechia; ^3^Department of Physiology, Faculty of Medicine in Pilsen, Charles University, Pilsen, Czechia; ^4^Third Department of Surgery, University Hospital Motol and First Medical School, Charles University, Prague, Czechia; ^5^Department of Anesthesia and Intensive Care Medicine, Faculty of Medicine in Pilsen, Charles University, Pilsen, Czechia; ^6^Department of Histology and Embryology, Faculty of Medicine in Pilsen, Charles University, Pilsen, Czechia

**Keywords:** sepsis, septic shock, acute organ dysfunction, mesenchymal stem cells, cell therapy, immunomodulation

## Abstract

**Background:** Treatment with mesenchymal stem cells (MSCs) has elicited considerable interest as an adjunctive therapy in sepsis. However, the encouraging effects of experiments with MSC in rodents have not been adequately studied in large-animal models with better relevance to human sepsis.

**Objectives:** Here, we aimed to assess safety and efficacy of bone marrow-derived MSCs in a clinically relevant porcine model of progressive peritonitis-induced sepsis.

**Methods:** Thirty-two anesthetized, mechanically ventilated, and instrumented pigs were randomly assigned into four groups (*n* = 8 per group): (1) sham-operated group (CONTROL); (2) sham-operated group treated with MSCs (MSC-CONTROL); (3) sepsis group with standard supportive care (SEPSIS); and (4) sepsis group treated with MSCs (MSC-SEPSIS). Peritoneal sepsis was induced by inoculating cultivated autologous feces. MSCs (1 × 10^6^/kg) were administered intravenously at 6 h after sepsis induction.

**Results:** Before, 12, 18, and 24 h after the induction of peritonitis, we measured systemic, regional, and microvascular hemodynamics, multiple-organ functions, mitochondrial energy metabolism, systemic immune-inflammatory response, and oxidative stress. Administration of MSCs in the MSC-CONTROL group did not elicit any measurable acute effects. Treatment of septic animals with MSCs failed to mitigate sepsis-induced hemodynamic alterations or the gradual rise in Sepsis-related organ failure assessment scores. MSCs did not confer any protection against sepsis-mediated cellular myocardial depression and mitochondrial dysfunction. MSCs also failed to modulate the deregulated immune-inflammatory response.

**Conclusion:** Intravenous administration of bone marrow-derived MSCs to healthy animals was well-tolerated. However, in this large-animal, clinically relevant peritonitis-induced sepsis model, MSCs were not capable of reversing any of the sepsis-induced disturbances in multiple biological, organ, and cellular systems.

## Introduction

The lack of effective therapy for sepsis remains a major unmet medical need. Even though substantial progress has been made in understanding the underlying pathophysiology of sepsis, translation of these advances into clinically effective therapies has been disappointing. Given the extreme complexity of sepsis pathogenesis, the paradigm “one disease, one drug” is obviously flawed and combinations of multiple targets that involve early immunomodulation and cellular protection are needed. The immunomodulatory, anti-inflammatory, anti-apoptotic, metabolomic, and anti-microbial effects of mesenchymal stem cells (MSCs) may have scientific and clinical relevance in this context (1). Indeed, application of MSCs in preclinical models of sepsis has been associated with lower mortality, improved course of sepsis due to inhibition of pro-active elements of the immune system, and a change in the pro- and anti-cytokine ratio both in *vitro* and *in vivo* (1–3). In addition, no preclinical study published so far has demonstrated adverse effects associated with the application of MSCs in animal models of sepsis. It must be emphasized, however, that these encouraging results were largely derived from rodent models with clearly limited relevance to human sepsis. Hence, a thorough investigation of the effects of MSCs in clinically relevant large-animal models is urgently needed before translation to the clinical field. Therefore, we conducted a randomized controlled experimental study to explore the biological effects of MSCs on the background of standard care in comparison to standard conservative therapy in a porcine model of peritonitis-induced progressive sepsis. The model fulfills recently defined requirements for preclinical sepsis studies (4). We aimed to examine both the safety of MSCs in healthy animals and the effect of MSCs on various biological systems related to multiple pathophysiological pathways during sepsis progression.

## Materials and Methods

### Mesenchymal Stem Cells

Allogenic porcine MSCs were isolated from healthy pigs. Bone marrow from the tibia or femur bones was aspirated into 50-mL tubes (Techno Plastic Products-TPP, Trasadingen, Switzerland) containing heparin (B Braun) by puncture with a sterile needle. MSCs were isolated from bone marrow by gradient centrifugation (440 × *g*, 30 min) on Ficoll-Paque Plus (GE Healthcare, North Richland Hills, Texas, USA). The layer of mononucleated cells was washed with phosphate-buffered saline (PBS) and plated in a 75-cm^2^ culture flask (TPP) containing α-MEM cell culture medium (Thermo Fisher Scientific, Waltham, MA, USA) supplemented with 10% fetal bovine serum (Thermo Fisher Scientific), 1 mM L-glutamine (Biochrom, Cambridge, UK), 6.0 mg/mL penicillin/10 mg/mL streptomycin (Biosera, Nuaille, France), and 0.25 mg/mL gentamicin (Biosera). Culture medium was changed every second day. After 10 days, MSCs were harvested by ethylenediaminetetraacetic acid (EDTA)/trypsin 1× (Biosera) and separated into three 75-cm^2^ culture flasks (TPP). Culture medium was changed again every second day, and after 10 days, MSCs were harvested by EDTA/trypsin 1× (Biosera) and cryopreserved in liquid nitrogen (1 × 10^6^ cells/cryotube). Four weeks before transplantation, MSCs were thawed, plated in 150-cm^2^ flasks (TPP) containing 20 mL of the culture medium described above, and cultured for 4 weeks to obtain about 5 × 10^7^ cells with one passage cycle. In this way, the stem cell properties of MSCs were maintained. On the day of transplantation, MSCs were harvested as described above, counted, re-suspended in 100 mL of saline solution (B Braun) pre-warmed to 37°C (10^6^/kg of pig weight) per pig, and immediately administered through the central venous line 6 h after induction of peritonitis. Before transplantation, the stem cell phenotype of MSCs was evaluated by flow cytometric detection of CD90, CD73, and CD44 markers (shown in Supplemental Digital Material). MSCs were washed with PBS and stained with 5 μL of APC-CD90 (Biolegend, San Diego, CA, USA), PE-CD73 (Biolegend), BV421-CD44 (Biolegend), and FITC-CD45 (Bio-Rad, Hercules, CA, USA) for 15 min in the dark at room temperature. Afterwards, MSCs were washed and resuspened in 300 μL of PBS followed by measurement on a BD FACS Aria Fusion cell analyzer (Becton Dickinson, Franklin Lakes, NJ, USA). Post-acquisition analysis of data was performed using FlowJo software (FlowJo LLC, Ashland, OR, USA). The ability of transplanted MSCs to differentiate was evaluated by their formation of adipo-, osteo-, and chondro-lineages. MSCs were seeded into 12-well-cultivation dishes (TPP) with a seeding density of 3.8 × 10^4^ cells/well for adipogenic and chondrogenic differentiation, and 1.9 × 10^4^ cells/well for osteogenic differentiation in culture medium. After a 24-h attachment period, the medium was discarded and replaced with 3 mL of StemPro® Adipogenesis Differentiation Kit, StemPro® Chondrogenesis Differentiation Kit, or StemPro® Osteogenesis Differentiation Kit (all Thermo Fisher Scientific) for adipogenic, chondrogenic, or osteogenic differentiation, respectively. After a differentiation period of 21 days, the cells were stained with oil red O for lipid droplet visualization in adipogenesis, alcian blue for glycoprotein visualization in chondrogenesis, and alizarin red S (all Sigma Aldrich, St. Louis, MO, USA) for calcium ion visualization in osteogenesis. Donor MSCs were not matched with recipients.

### Animals

All experiments were performed in adherence to the European Directive for the Protection of Vertebrate Animals Used for Experimental and Other Scientific Purposes (86/609/EU). The protocols were approved by the Committee for Experiments on Animals of the Faculty of Medicine, Charles University, in Pilsen and by the Ministry of Education, Youth and Sports of the Czech Republic (protocol no. MSMT-20064/2015-3). All experiments were performed in the Laboratory of Experimental Intensive Care Medicine of the Biomedical Center at the Faculty of Medicine in Pilsen. Thirty-two domestic pigs (breed Black Pied Prestice Pig) from conventional breeding facility (ZD Mladotice, Czech Republic) of either sex with a median weight of 43.5 (40–46) kg were used.

### Experimental Protocol

**Figure 1:**
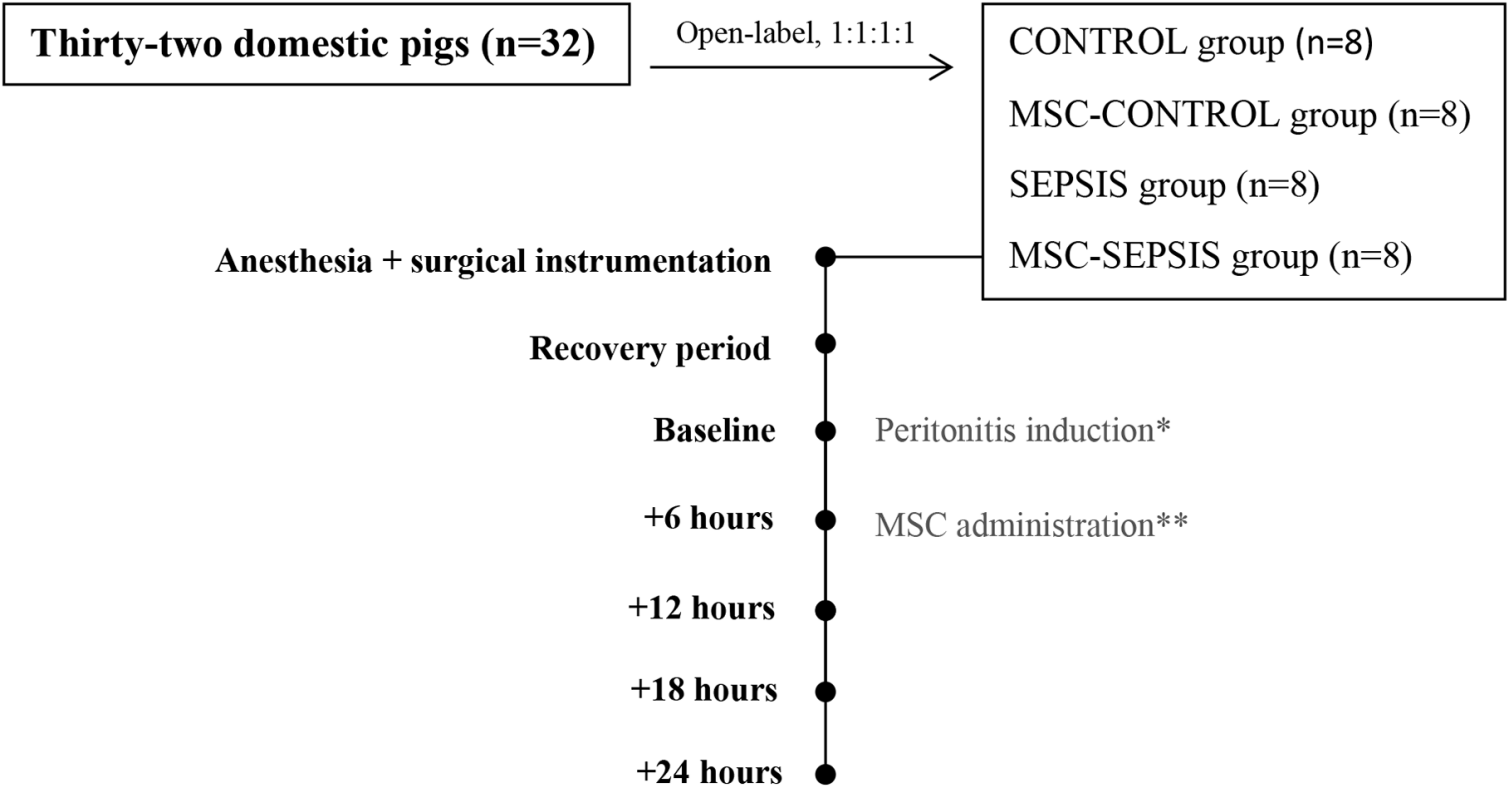
Basic scheme of experimental protocol. The (*) stands for peritonitis induction in septic groups (SEPSIS and MSC-SEPSIS). The (**) stands for MSCs administration in treated groups (MSC-CONTROL and MSC-SEPSIS).

The animals were assigned to one of four experimental groups (at a ratio of 1:1:1:1): sham-operated control group (CONTROL, *n* = 8), control group treated with MSCs (MSC-CONTROL, *n* = 8), sham-operated sepsis group (SEPSIS, *n* = 8), and septic group treated with MSCs (MSC-SEPSIS, *n* = 8). The intervention was open-labeled. In septic animals, peritonitis was induced by inoculating 1 g/kg of autologous feces (collected preoperatively and suspended in 200 mL of isotonic saline at 38°C) into the abdominal cavity followed by a 6-h recovery period (baseline). When sepsis-associated hypotension developed, fluid boluses (10 ml/kg of Ringerfundin solution) were administered in a goal-directed manner guided by filling pressures and cardiac output response as part of hemodynamic resuscitation. Fluid resuscitation was discontinued if there was no further increase in cardiac output (10% threshold) and/or when the pulmonary artery occlusion pressure (PAOP) reached more than 15 mmHg. Continuous infusion of norepinephrine was administered if the mean arterial pressure (MAP) fell below 65 mmHg and no further positive hemodynamic response was elicited via fluid resuscitation. Norepinephrine was titrated to maintain MAP between 65 and 70 mmHg. In MSC-CONTROL and MSC-SEPSIS groups, MCSs were infused in a clinically relevant dose (1 × 10^6^/kg) over 10 min via the central venous line 6 h from the baseline. The MSC dose was chosen on the basis of several previous clinical (5, 6) as well as experimental rodent (1, 2) and large animal studies (7). At the end of the experiment, the animals were euthanized by anesthetic overdose and excision of the heart. Experimental protocol scheme is shown in Figure 1.

### Anesthesia and Surgical Instrumentation

All animals were anesthetized with xylazine (1 mg/kg) and tiletamin-zolazepam (5 mg/kg). A 2 mg/kg dose of 2% propofol was administered after intravenous line insertion. Animals were intubated and mechanically ventilated as follows: volume control mode with tidal volume of 8–10 mL/kg, positive end-expiratory pressure of 0.6 kPa, FiO_2_ of 0.3, and respiratory rate adjusted to maintain arterial normocapnia. During surgery, anesthesia was maintained by continuous administration of 2% propofol (4–6 mg/kg/h), fentanyl (8–10 μg/kg/h), and rocuronium (2 mg/kg/h). Drug dosing was halved after surgery. Continuous infusion of Ringerfundin solution (B-Braun Melsungen AG, Melsungen, Germany) was used as a fluid replacement at a dose of 10 mL/kg/h during surgery and 7 mL/kg/h thereafter. Continuous infusion of 10% glucose served to maintain arterial blood normoglycemia.

Before the surgical procedure, an arterial catheter was placed in the femoral artery for continuous invasive blood pressure monitoring and blood sampling. Pulmonary artery and central venous catheters were introduced via external jugular veins under ultrasound guidance. Midline laparotomy was performed and a pre-calibrated ultrasound flow probe (Transonic Systems, Ithaca, NY, USA) was placed around the left renal artery. Double-lumen ileostomy was constructed to assess gut mucosal microcirculation. Peritoneal drainage was inserted and epicystostomy was performed prior to abdominal wall closure. A recovery period of 6 h followed the surgical procedures.

### Monitoring, Sampling, and Measurements

Data sets were recorded at baseline/sepsis induction, +12, +18, and +24 h after peritonitis induction. Measurements and calculations included the assessment of systemic and regional hemodynamics (see Supplemental Digital Material), fluids, and vasopressor requirement. Arterial blood samples were analyzed for lactate, arterial and mixed venous blood gases, pH, base excess by using POCT analyzer (Cobas B 123, Roche, Diagnostics, USA). Complete blood count and other biochemical analyses included serum creatinine, aspartate aminotransferase (AST), alanine aminotransferase (ALT), high-sensitivity troponin I (hsTnI), albumin, and total protein were performed as described previously (8). Cytokines, including IL-6, IL-8, IL-10, and tumor necrosis factor α (TNF-α), as well as C-reactive protein were analyzed by using the ELISA method (Porcine Quantikine ELISA Kit, R&D System, Minneapolis, USA). Oxidative stress biomarker 8-isoprostane analysis was performed by using EIA porcine kit (Cayman Chemical, Michigan, USA). In addition, basic hemodynamics and acid-base balance parameters were measured prior to MSC's administration (i.e., + 6 h, data not shown) to capture sepsis development.

The modified sepsis-related organ failure assessment (SOFA) score was determined according to the Third international consensus definitions for sepsis and septic shock (9) with exclusion of the Glasgow coma scale-based neurologic component. Gut mucosal microcirculation was recorded at each time point using a MicroScan handheld video microscope (MicroVision Medical, Amsterdam, Netherlands). Each record was split into three parts to visualize different areas of the gut mucosa and analyzed by Automated Vascular Analysis software version 4.0 (MicroVision Medical).

Cardiac myocytes were isolated from the left ventricle by enzymatic dissociation with collagenase A (Sigma-Aldrich) as previously reported (8). Sarcomeric contractions of isolated cardiac myocytes were measured with the HyperSwitch Myocyte Calcium and Contractility System (IonOptix LLC, Westwood, MA, USA), with the Sarclen sarcomere length acquisition module. Measurements were performed in normal Tyrode solution at 37 ± 0.5°C. Cells were stimulated with the MyoPacer Field Stimulator (IonOptix LLC) at cycles of 300, 500, 1,000, and 2,000 ms. IonWizard 6.5 software (IonOptix LLC) was used for offline analysis.

Cardiac mitochondrial function was assessed using high-resolution respirometry (oxygraph Oroboros O2k; Oroboros Instruments, Innsbruck, Austria). Mitochondrial oxygen consumption was measured in permeabilized left ventricular samples at 37°C. Transmural sample (~1 cm^3^) was dissected from the proximal free wall of the left ventricle and cut into 2 mg tissue samples that were quickly transferred into ice-cold biopsy preserving solution (BIOPS: 10 mM Ca-EGTA buffer, 0.1 μM free calcium, 20 mM imidazole, 20 mM taurine, 50 mM K-MES, 0.5 mM DTT, 6.56 mM MgCl2, 5.77 mM ATP, 15 mM phosphocreatine, pH 7.1) with saponin (50 μg/ml), shaken gently on ice for 30 min, washed in mitochondrial respiration medium (MiR06: 0.5 mM EGTA, 3 mM MgCl2.6H2O, 60 mM lactobionic acid, 20 mM taurine, 10 mM KH2PO4, 20 mM HEPES, 110 mM D-sucrose, 1 g/l albumin essentially fatty acid free, and 280 u/ml catalase) for 10 min and then placed into oxygraph chambers. In the titration protocol, several substrates and inhibitors of the mitochondrial respiratory system were sequentially added into the chambers to determine particular respiratory states and activities of mitochondrial respiratory complexes. Oxygen consumption was analyzed online by DatLab software (Oroboros Instruments) as the negative time derivative of oxygen concentration in the chamber.

### Flow Cytometry of Leukocytes Subpopulations

**Figure 2:**
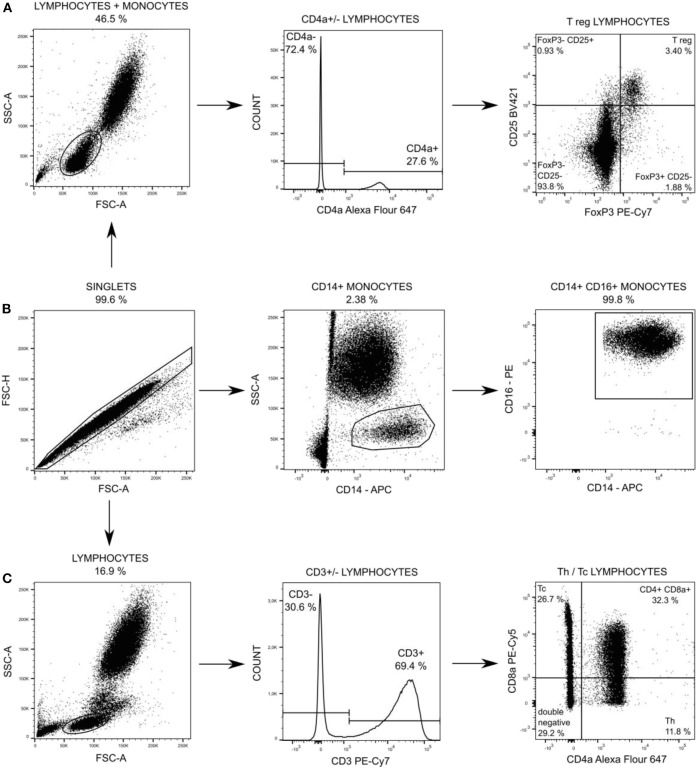
Particular subpopulations of leukocytes were gated from singlets populations followed by gating on FSC and SSC **(A,C)** and followed by gating on specific T regulatory (T reg) lymphocytes **(A)**, on CD14+ CD16+ monocytes **(B)**, and on T-helper (Th) lymphocytes and cytotoxic (Tc) + CD8α+ γδ T lymphocytes **(C)**.

Changes in leukocytes subpopulations were monitored by flow cytometry. One hundred microliter of EDTA treated blood was stained for 15 min in dark and room temperature by cocktail of anti-CD specific antibodies (Table antibodies, Supplemental Digital Material) at baseline, 12 and 18 h after peritonitis induction (24 h was not measured due to operational reasons). Afterward staining, all samples were lysed by BD FACS Lysing Solution (Becton Dickinson, San Jose, USA) to separate leukocytes and contaminating erythrocytes. CD14posCD16pos monocytes, T-helper (Th) and cytotoxic T (Tc) + CD8α+ γδ T lymphocytes were washed by PBS, pelleted (300 × g, 5 min) and re-suspened in 300 μl of PBS followed by measurement. T regulatory (Treg) lymphocytes were fixed and permeabilized by FoxP3 staining buffer set (Thermo Fisher Scientific), washed by PBS, pelleted (300 × g, 5 min) and re-suspened in 300 μl of PBS followed by measurement. The measurement was performed by the BD FACS Aria Fusion cell analyzer (Becton Dickinson). One million events was acquired and the post-acquisition analysis of data was performed using FlowJo software (Becton Dickinson). The gating strategy for each subpopulation is summarized in Figure 2. Absolute counts of particular subpopulations were determined from total leukocytes counts acquired within standard biochemical analysis of blood samples.

### Statistical Analysis

Statistical analysis was carried out using SigmaStat software version 3.5 (Systat Software Inc., Erkrath, Germany). Results are presented as median (interquartile range, range). Statistical comparisons were made using non-parametric statistics. Differences within each group before and after induction of sepsis were tested using Friedman ANOVA on ranks and, subsequently, Dunn's test for multiple comparisons. The Mann-Whitney rank sum test was performed to compare data between treatment groups (CONTROL vs. MSC-CONTROL; SEPSIS vs. MSC-SEPSIS). A *p* < 0.05 was regarded as statistically significant.

## Results

**Figure 3:**
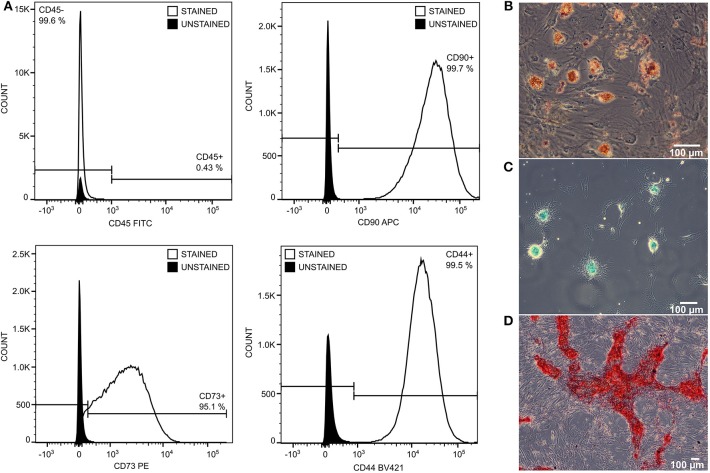
The quality of transplanted MSCs was monitored by expression of stem cell surface markers and by differentiation ability. MSCs were negative to CD45 and positive to CD90, CD73, and CD44 **(A)**. They differentiate into adipo- **(B)**, chondro- **(C)**, and osteo- **(D)** lineage in 21 days of differentiation protocol.

Administrated MSCs achieved standard quality as determined by the expression of stem cell markers CD90^+^, CD73^+^, CD44^+^, CD45^−^, and by their ability to differentiate into adipo-, chondro-, and osteo-lineages (Figure 3).

Five experiments were repeated due to perioperative death (*n* = 2) or premature death before baseline data collection or treatment commencement (*n* = 3). Data from animals that died prematurely were not used for analysis. Administration of MSCs to sham-operated healthy animals did not induce any significant alterations in systemic, regional, or microvascular hemodynamics (data not shown). Similarly, neither target organ functions nor markers reflecting inflammatory status and oxidative stress were affected by MSCs (Table 1).

**Table 1:** Parameters describing organ function and systemic inflammation in sham-operated animals with and without MSC administration.

**Parameter**	**Timepoint**	**CONTROL**	**MSC-CONTROL**
Urea (mmol/L)	Baseline	5 (4.2–5.5)	5.7 (5–6.6)
	+12 H	5.5 (3.6–5.9)	6.1 (5.1–7.3)^*^
	+18 H	5 (3.3–5.6)	5.5 (4.5–6.6)
	+24 H	4.4 (3.2–4.6)	4.7 (3.8–5.8)^*^
Creatinine (μmol/L)	Baseline	95 (80–103)	99 (87–110)
	+12 H	93 (77–108)	100 (88–107)
	+18 H	88 (77–102)	95 (86–102)
	+24 H	85 (68–93)	89 (83–103)
AST (μkat/L)	Baseline	0.7 (0.6–1.5)	0.8 (0.6–1.9)
	+12 H	0.8 (0.7–1.4)	0.9 (0.8–1.1)
	+18 H	0.8 (0.7–1.6)	1.0 (0.9–1.5)
	+24 H	0.8 (0.7–1.4)	1.0 (0.9–1.4)
ALT (μkat/L)	Baseline	0.5 (0.5–0.7)	0.6 (0.5–0.7)
	+12 H	0.5 (0.5–0.6)	0.5 (0.48–0.51)^*^
	+18 H	0.5 (0.4–0.6)	0.5 (0.47–0.50)^*^
	+24 H	0.5 (0.4–0.5)^*^	0.52 (0.5–0.5)^*^
Trombocytes (1 × 10^9^/L)	Baseline	285 (226–315)	260 (231–357)
	+12 H	227 (204–302)	200 (191–236)^*^
	+18 H	236 (213–271)	201 (164–220)^*^
	+24 H	241 (216–252)	194 (190–226)^*^
PaO_2_/FiO_2_ (mmHg)	Baseline	459 (410–510)	450 (441–466)
	+12 H	408 (399–460)	452 (410–455)
	+18 H	344 (262–404)^*^	355 (327–414)^*^
	+24 H	272 (232–353)^*^	313 (187–412)^*^
hsTnT (ng/L)	Baseline	7.2 (6.2–18.1)	9.7 (7.4–36.3)
	+12 H	7.7 (6.7–21.6)	9.2 (7.6–24.8)
	+18 H	6.1 (5.6–17.4)^*^	9 (7.1–19.9)
	+24 H	5.9 (4.5–17.8)^*^	10.5 (6.6–18.7)
IL-6 (ng/L)	Baseline	47 (36–75)	59 (47–114)
	+12 H	48 (30–63)	38 (16–64)
	+18 H	64 (48–212)	61 (33–66)
	+24 H	352 (139–1,451)^*^	175 (35–768)
TNF-α (ng/L)	Baseline	91.3 (77.7–123.2)	100.3 (62.1–145.1)
	+12 H	58.9 (54.2–89.5)	52.7 (38.8–75.8)^*^
	+18 H	60.0 (53.4–68.5)	59.3 (50.7–88.9)^*^
	+24 H	76.5 (61.5–119.3)	94.2 (57.4–112.2)
8-Isoprostane (μg/L)	Baseline	32.0 (4.9–55.8)	43.3 (23.0–93.2)
	+12 H	8.4 (6.7–28.5)	23.5 (11.6–31.3)
	+18 H	5.7 (4.9–14.1)	15.1 (4.0–37.1)
	+24 H	6.7 (4–72.3)	55.6 (14.1–89.4)

*AST, aspartate aminotransferase; ALT, alanine aminotransferase; PaO_2_/FiO_2_, oxygenation index; hsTnT, high-sensitivity troponin T; IL-6, interleukin 6; TNF-α, tumor necrosis factor α*.

* *p < 0.05 between baseline and time-point. No statistical significance was found between groups*.

All animals in both septic groups developed sepsis according to SEPSIS-3 criteria. Seven animals in the sepsis group and six animals in the MSC-SEPSIS group completed the whole 24-h protocol. Three animals (*n* = 1 in sepsis, *n* = 2 in MSC-SEPSIS group) died prematurely due to refractory septic shock. At the start of treatment (i.e., 6 h after induction of peritonitis) there were no statistically significant differences in any measured variables between SEPSIS and MSC-SEPSIS groups (data not shown).

**Figure 4:**
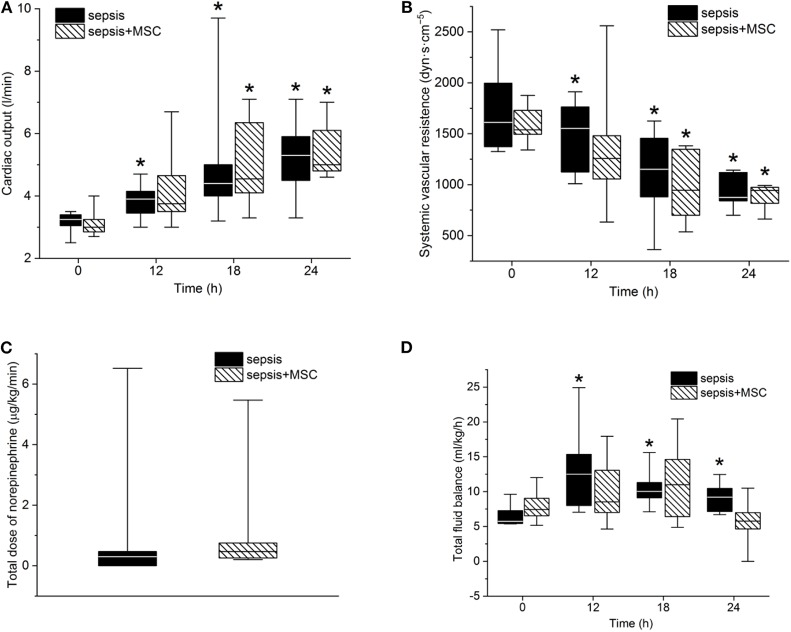
**(A)** Cardiac output, systemic vascular resistance **(B)**, total dose of norepinephrine **(C)** and total fluid balance **(D)**. The (*) stands for significant difference in time (*p* < 0.05 vs. baseline).

After the induction of peritonitis, all pigs developed hyperdynamic sepsis with an increased cardiac output and reduced systemic vascular resistance, without intergroup differences (Figures 4A,B). Six pigs (75%) in the sepsis group and eight pigs (100%) in the MSC-SEPSIS group required vasopressor support to maintain MAP above 65 mmHg. The total dose of norepinephrine was comparable in both septic groups (Figure 4C) as was the time to the first administration of norepinephrine [1,093 (885–1,165) min in SEPSIS vs. 748 (594–944) min in MSC-SEPSIS group; *p* = 0.345]. Likewise, there were no significant differences in the amount of fluid administered for hemodynamic resuscitation (Figure 4D). Other hemodynamic and metabolic variables are summarized in Table 2.

**Table 2:** Hemodynamic and metabolic variables in septic animals with and without MSC administration.

**Parameter**	**TP**	**SEPSIS**	**MSC-SEPSIS**
MAP (mmHg)	Baseline	73 (68–81)	70 (64–74)
	+12 H	80 (70–87)	69 (66–75)
	+18 H	71 (64–71)	68 (64–73)
	+24 H	72 (69–73)	72 (68–75)
PAOP (mmHg)	Baseline	9 (8–9)	9 (6–10)
	+12 H	11 (8–11)	8 (6–10)
	+18 H	11 (10–13)	8 (7–10)
	+24 H	10 (9–12)	9 (8–11)
CVP (mmHg)	Baseline	8 (8–10)	7 (7–9)
	+12 H	11 (10–12)^*^	9 (8–12)
	+18 H	12 (10–14)^*^	10 (8–14)^*^
	+24 H	11 (10–15)^*^	12 (10–14)^*^
DO_2_ (mL/min/kg)	Baseline	10.2 (9.1–11.0)	10.2 (9.0–10.7)
	+12 H	12.2 (11.2–15.3)	14.4 (12.7–16.2)
	+18 H	15.1 (12.4–17.9)	16.6 (14.8–19.5)
	+24 H	16.7 (14.9–21.0)^*^	20.5 (18.7–26.5)^*^
VO_2_ (mL/min/kg)	Baseline	5.3 (4.5–5.6)	5.1 (4.7–5.6)
	+12 H	5.7 (4.9–6.4)	6.0 (5.4–6.8)
	+18 H	5.7 (5.3–6.3)	6.7 (5.6–7.7)
	+24 H	6.1 (5.7–6.4)	6.8 (6.6–8.1)^*^
RBF/CO (%)	Baseline	6.7 (5.5–10.5)	6.2 (5.4–7.5)
	+12 H	5.3 (3.5–6.3)^*^	4.0 (2.2–5.0)
	+18 H	4.6 (2.9–5.6)^*^	2.2 (0.9–4.1)^*^
	+24 H	3.7 (2.1–5.4)^*^	3.0 (2.2–3.7)^*^
Total vessel density (gut mucosa) (mm/mm^2^)	Baseline	16.0 (14.5–22.9)^#^	26.6 (22.6–28.7)^#^
	+12 H	22.2 (13.1–24.2)	20.2 (16.0–32.8)
	+18 H	21.4 (16.6–26.0)	19.4 (17.6–22.2)
	+24 H	17.4 (12.0–27.6)	17.7 (15.1–23.6)
Perfused vessel density (gut mucosa) (mm/mm^2^)	Baseline	15.9 (14.5–22.9)	26.6 (18.7–28.7)
	+12 H	16.8 (12.5–20.3)	20.1 (16.0–31.6)
	+18 H	20.6 (15.7–26.0)	18.3 (16.5–20.8)
	+24 H	17.4 (11.8–27.6)	16.9 (15.1–22.1)
Arterial base excess (mmol/L)	Baseline	4.9 (3.6–5.7)	4.0 (2.8–5.2)
	+12 H	0.8 (−0.2–1.7)^*^	0.2 (-0.9–1.5)
	+18 H	−0.1 (−1.6–1.7)^*^	−0.6 (-9.8–0.1)^*^
	+24 H	−1.5 (−2.7–2.4)^*^	−1.7 (-3.4–1.0)^*^
Arterial lactate (mmol/L)	Baseline	1.1 (1.0–1.2)	1.1 (1–1.2)
	+12 H	1.1 (1.0–1.2)	1 (1–1.1)
	+18 H	1.2 (1.1–1.3)	1.3 (1–5.5)
	+24 H	1.5 (1.0–2.4)	1.5 (1.3–1.8)
Hemoglobin (g/dL)	Baseline	10.1 (9.7–11.1)	9.9 (8.9–10.5)
	+12 H	10.9 (10.5–12.2)^*^	11.6 (10.4–12)^*^
	+18 H	11.2 (10.8–12.4)^*^	11.2 (9.6–12.6)^*^
	+24 H	11.9 (10.5–12.4)^*^	12.7 (12.2–12.8)^*^

*MAP, mean arterial pressure; PAOP, pulmonary artery occlusion pressure; CVP, central venous pressure; DO_2_, systemic oxygen delivery; VO_2_, systemic oxygen uptake; RBF, renal artery blood flow; CO, cardiac output*.

# p < 0.05 between SEPSIS and MSC-SEPSIS group;

* *p < 0.05 between time-point and baseline*.

**Figure 5:**
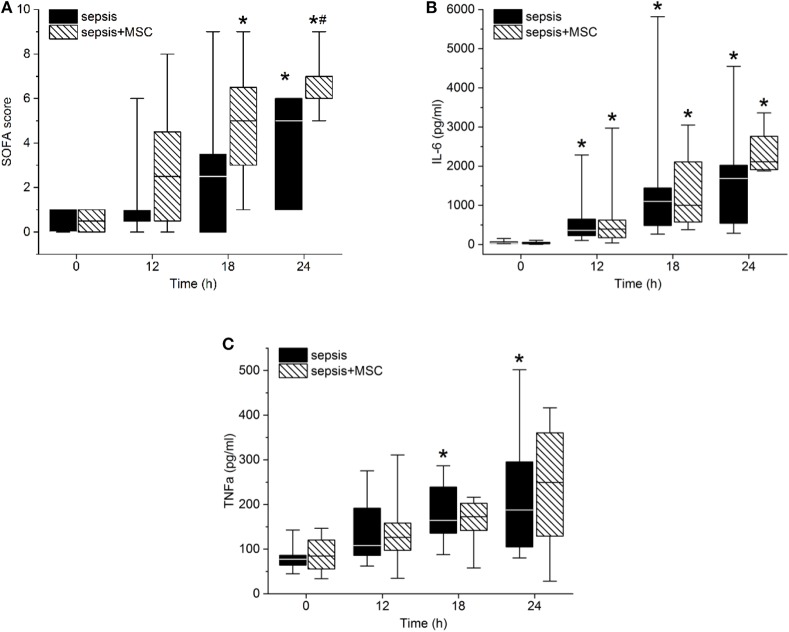
Sepsis-related organ failure assessment (SOFA) score **(A)**, Interleukin-6 **(B)**, and TNFα serum levels **(C)**. The (#) stands for statistical significance between the groups (*p* < 0.05). The (*) stands for significant differences in time in particular groups (*p* < 0.05 vs. baseline).

The modified SOFA score progressively increased in both septic groups. Treatment with MSCs failed to attenuate sepsis-induced organ dysfunction. The tendency of the SOFA score to increase was even more pronounced in the MSC-SEPSIS group, mainly as a result of earlier initiation of norepinephrine administration (Figure 5A). Indeed, no sign of a beneficial effect of MSCs was observed even when single organ systems included in SOFA score (i.e., lungs, kidneys, liver, platelets) were evaluated separately (single organ data not presented).

**Figure 6:**
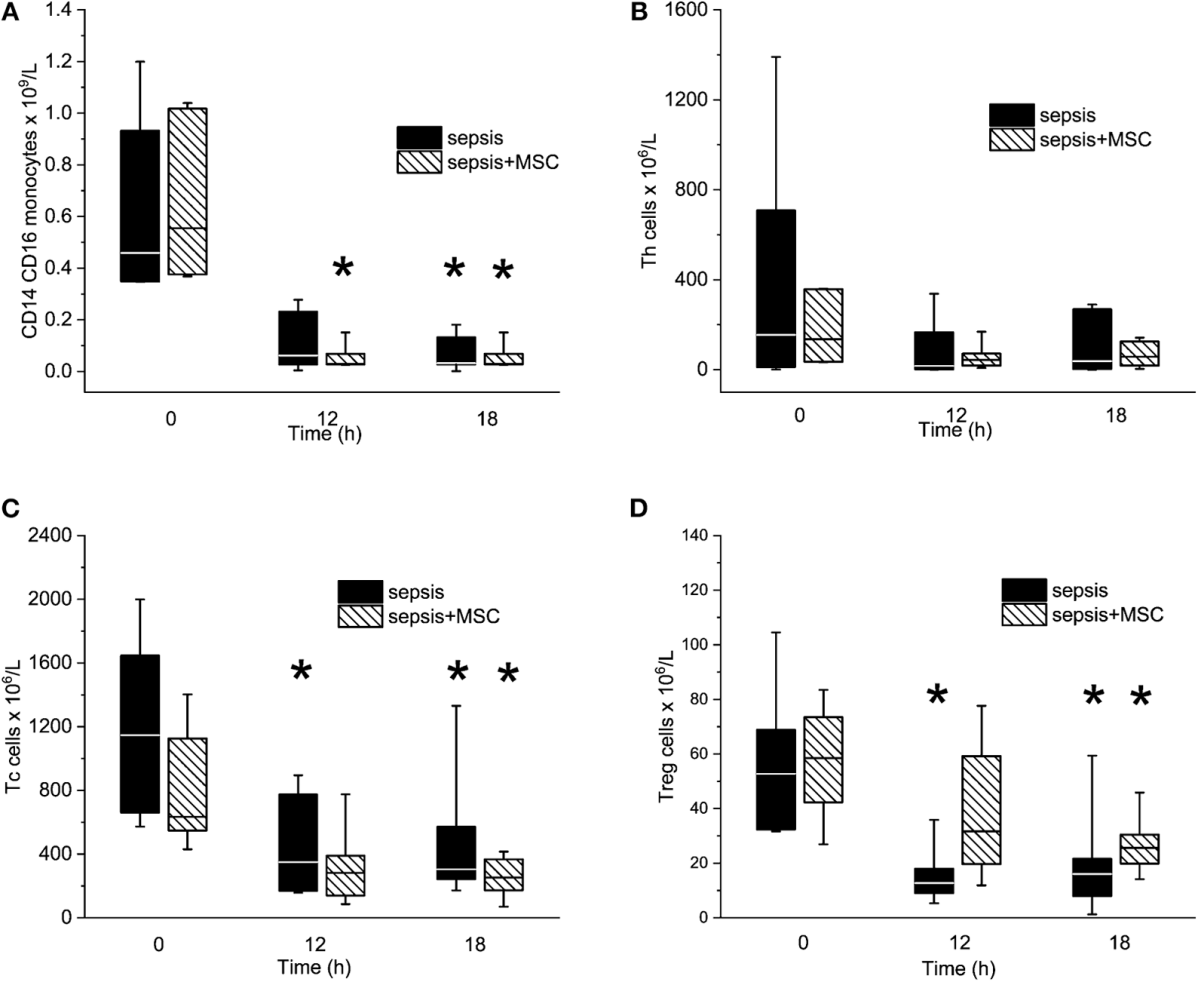
The absolute numbers of CD14/CD16^pos^ monocytes **(A)**, T helper lymphocytes **(B)**, Tc + CD8α+ γδ T lymphocytes **(C)**, and T regulatory lymphocytes **(D)**. The (*) stands for significant difference between particular time point and baseline only (*p* < 0.05).

Peritonitis-induced sepsis resulted in gradually increased plasma levels of TNF-α and IL-6, providing evidence of a progressive systemic inflammatory response (Figures 5B,C). Treatment with MSCs did not result in a favorable effect on any of these variables. Plasma levels of IL-10 remained under the detection limit in all animals. Levels of CD14^+^ CD16^+^ monocytes, and Th, Tc + CD8α+ γδ T lymphocytes, and T_reg_ lymphocytes decreased in time due to sepsis in both experimental groups. The CD3+CD4a–CD8a+CD8b+gating consistently provided lower cell numbers than the CD3+CD4a–CD8a+gating [50–70%, e.g., 69% (34%, 63%) in sepsis baseline, 62% (36%, 66%) in 18 h sepsis vs. 48% (36%, 64%) in sepsis+MSC baseline, 60% (42%, 63%) in 18 h sepsis+MSC] suggesting a significant population of TCR γδ cells. Importantly, the application of MSCs caused no significant differences when comparing SEPSIS and MSC-SEPSIS groups at each time point (Figure 6). Data on full blood count and other inflammatory parameters in sham-operated and septic animals with and without MSC administration are shown in Supplemental Digital Material (Tables 1, 2).

**Figure 7:**
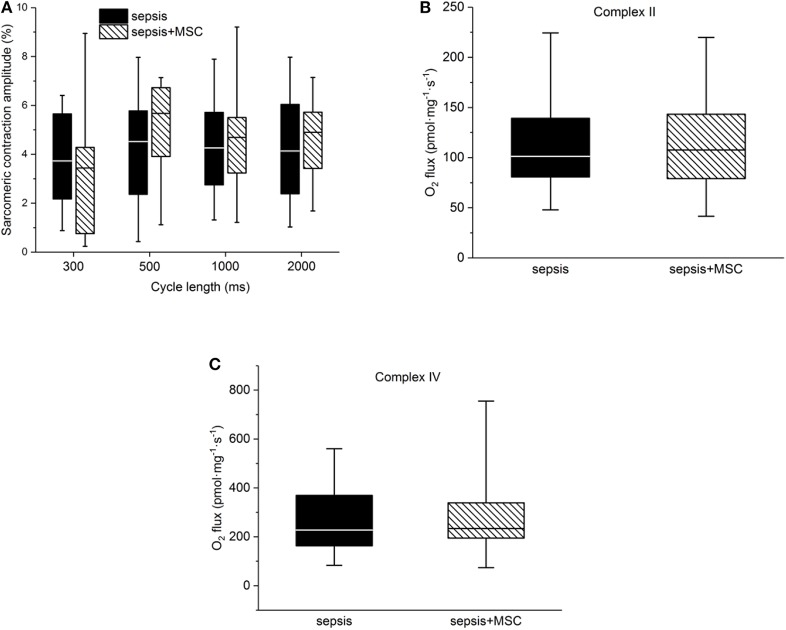
Cellular myocardial depression. **(A)** Relative sarcomeric contraction amplitudes in cardiac myocytes from septic animals with or without application of MSC. **(B)** Mitochondrial respiration. Complex II-dependent oxygen consumption in septic myocardium without or with application of MSC. **(C)** Mitochondrial respiration. Complex IV-dependent oxygen consumption in septic myocardium without or with application of MSC.

In isolated cardiac myocytes, sarcomeric contractions were decreased in septic cells at lower stimulation rates (1, 0.5 Hz). Application of MSC did not exert any significant effect on sarcomeric contraction regardless whether in control (non-septic; not shown) or septic cardiac myocytes (Figure 7A). Kinetic parameters of sarcomeric contractions (e.g., time to 50% of peak contraction, time to 50% relaxation) were not affected by sepsis nor by application of MSCs (not shown). Mitochondrial respiration was suppressed in septic hearts and this reduction was mainly due to inhibition of Complex II and IV. Application of MSCs, either in control (not shown) or septic animals, did not affect cardiac mitochondrial respiration (Figures 7B,C).

## Discussion

The present study was designed to evaluate the short-term safety, tolerability, and efficacy of a single intravenous administration of bone marrow-derived MSCs in a large-animal, peritonitis-induced sepsis model. The model was characterized by the development of the full spectrum of sepsis-induced organ dysfunction with typical hemodynamic, metabolic, and inflammatory host response phenotypes. The main findings indicate that: (1) the application of MSCs to healthy animals was well-tolerated without any measurable acute effects on macro- and microcirculatory hemodynamics, organ, and mitochondrial functions; (2) early treatment with MSCs failed to mitigate the development of sepsis-induced hemodynamic alterations including the progression of sepsis to septic shock; (3) MSCs did not confer any protection against alterations in cellular energy metabolism and multiple organ functions; and (4) treatment failed to counteract a gradual sepsis-driven systemic immune-inflammatory response.

The discrepancy between the salutary effects of MSCs reported in multiple preclinical models (10–15) and the apparent absence of any sign of improvement in multiple biological systems in this model is a striking and key finding of this study, deserving critical discussion. Many emerging treatment strategies shown to be effective in preclinical studies, generally failed to yield beneficial effects in clinical trials. Numerous arguments have been proposed to explain the failure to translate experimental results into effective treatments for human sepsis, including complexity and heterogeneity of sepsis, methodologically inappropriate clinical trials, and clinically irrelevant animal models (4, 16). The latter may prove particularly important in the context of our study. Most notably, all but one study published thus far have involved exclusively rodents, mainly mice (1, 2, 7). The marked difference in the immune-inflammatory response to insults between rodents and humans is well-documented (17). Fundamental differences include, but are not limited to, the divergence of the transcriptomic response, the mismatch of temporal response patterns, differences in both innate and adaptive immunity, and/or the homogeneity of highly inbred mouse strains (18). Pigs, on the other hand, show very similar endotoxin sensitivity and tissue antigenicity, similar cardiovascular and renal physiology including hyperdynamic circulation in sepsis and similar temporal response pattern to humans (19). Additional technical advantages are associated with bigger body size that is comparable to humans and allows extensive instrumentation, continuous monitoring, and serial blood sampling. Moreover, frequent use of specific pathogen-free (SPF) animals in sepsis research, where alterations of the gut microbiome may markedly alter the animal's immune and inflammatory functions and susceptibility to infection, may also contribute to the disconnect between animal studies showing promising drug development and failure to translate to humans. In an interesting recent study a more straightforward comparison of response to anesthesia and surgical trauma was made between conventional and SPF rats (20). Comparison between conventional and SPF animals within one species and even strain (Spraque-Dawley) revealed decreased tolerance to anesthesia, hemodynamic instability, aberrant hematology, traumatic bleeding, and reduced physiological reserve in SPF animals. This altered phenotype to the stress of surgical trauma was completely reversed when SPF animals were returned to the original conventional facility. The role of gut microbiota is another aspect worth consideration when discussing factors potentially affecting the host response to infectious stimulus. It has been demonstrated that variations in the gut microbiota of donor mice influenced clinical as well as molecular phenotype of sepsis (21). At the time of our study, we did not assess individual microbial composition of porcine feces. However, given the single provider of laboratory animals with identical environmental conditions and comparable individual hemodynamic, metabolic, and inflammatory responses to feces, we hypothesize that the putative role of inter-individual variations in fecal microbiota was rather limited in this experiment. Taken together, the genetic and physiological proximity of pigs and humans makes this species an excellent biomodel for translational research, and utilization of domestic (farm) pigs exposed to similar environmental pathogens as humans is clearly superior to mouse models, in which the SPF handling probably limits the clinical translatability even more fundamentally (22, 23).

Our results are also in sharp contrast with data from Laroye et al., who reported less profound alterations in systemic hemodynamics and, therefore, longer time to death in septic pigs treated with human umbilical cord-derived MSCs (7). Surprisingly, the hemodynamic improvement observed in the above study occurred despite the lack of clear evidence for significant effects of MSCs on inflammation, bacterial clearance, and a rather transient alleviation of lung and kidney function. Although the dose and timing of interventions with MSCs were comparable to those reported here, the two studies differed with respect to the source of MSCs. As the secretome of umbilical cord-derived MSCs used by Laroye et al. might differ from bone marrow-derived MSCs, it is possible that MSCs from different sources possess different immunomodulatory capacity (24, 25). Bone marrow-derived MSCs have not only been proven to be effective in several small-animal sepsis studies (10, 24, 26), but they are currently the preferred source of MSCs in clinical settings and the only one with the ability to restore the original niche (27). Nevertheless, in a mouse model of endotoxin-induced sepsis, MSCs ameliorated sepsis-associated organ injury and mortality in spite of different MSC sources (26). Furthermore, the infectious burden and severity of sepsis were three times higher in Laroye's experiment compared to our study and the time course of pro-inflammatory cytokines, in particular TNF-α, markedly differed from our model, thus suggesting a different inflammatory environment. Whether these differences may have accounted for such discrepancy in results remains speculative.

Understanding existing methodological limitations and obstacles is essential for further translational research. Even though they were used successfully in previous experiments (28), we investigated the effects of one dose and one source of MSCs. Furthermore, MSCs were administered at a single time point. It would be of interest to evaluate the efficacy of MSCs obtained from different tissue sources, given in a dose-response fashion, and administered at different time points within the same experimental setting. The timing may be of critical importance for the interpretation of our results, because in the vast majority of positive preclinical studies, MSCs were administered very early, i.e., within 4 h after sepsis induction (2). No study has tested the application of MSCs beyond a 6-h time window (2). Indeed, the potential role of timing of the intervention has recently been demonstrated in a human endotoxemia model (29). In that study, intravenous infusion of allogenic human adipose MSCs exerted mixed time-dependent pro-inflammatory or anti-inflammatory and pro-coagulant effects (28). This suggested considerable biological complexity of MSCs and, possibly, a relatively narrow time frame for the treatment of early sepsis. Another relevant issue is the role of antibiotics in potentiating the action of MSCs. In a mouse model of sepsis induced by cecal ligation and puncture, combined treatment with MSCs and antibiotics greatly improved sepsis-associated symptoms and survival, indicating some synergistic effect (30). In our study, antibiotic therapy was not used. Our model was designed to create hyperdynamic sepsis, with increasing severity over time. Antibiotic therapy was expected to blunt the host response, thereby attenuating the development and full manifestation of a true clinical septic response during 24 h, which is what we instead sought to elicit in our experiment. Nevertheless, the interactions between antibiotic therapy and MSCs represent another important question warranting further investigation. We cannot exclude that the protective effect of MSCs might have manifested through other markers/pathways not monitored in our study. However, given that we assessed several clinically relevant and mutually independent biological targets, including macro- and microcirculatory perfusion, multiple organ functions, mitochondrial energy metabolism, systemic immune-inflammatory response, and oxidative stress, it seems unlikely that we would have missed major treatment effects.

The manufacturing of MSCs for this study was consistent with procedures applied elsewhere using fetal bovine serum for MSC propagation, trypsin for MSC harvesting, and a single freeze/thaw cycle for MSCs cryopreservation (10, 11, 15). Transplanted MSCs conformed to the criteria of the International Society for Cellular Therapy (31) and were kept for the minimum number of passages (up to four) to maintain their stem cell phenotype and avoid senescence (32). Paradoxically, the inconsistency in MSCs potency could lie in their sensitivity to a cytokine environment (33). As each pathology and even patient serum is accompanied by specific qualitative and quantitative cytokine composition, the immunomodulatory function of MSCs can be highly dependent on a particular patient's response to a specific pathology or treatment.

Another issue with possible role in MSCs potency is their matching to the recipient. There is the lack of studies comparing effects of matched vs. mismatched MSCs in the large animal model of sepsis. However, the clinical study of the team of Garcia-Sancho (34) showed that better HLA matching of donor MSCs with recipients did not enhance the efficacy of MSCs therapy in osteoarthritis and degenerative disc disease. Furthermore, there are several completed clinical trials applying allogenic MSC without any analysis of donor MSCs and recipient matching (5, 35–37) indicating that the donor MSCs and recipient matching may not play the role in MSCs potency. This direction is further supported by tendencies to apply pooled MSCs batches as better available and reproducible source of MSCs (38). However, upon the lack of available data comparing matched vs. mismatched MSCs therapy in sepsis relevant animal models we can only speculate about the role of this issue in MSCs efficacy in general.

Despite these limitations, our study benefits from a number of strengths and important findings. The history of sepsis research has repeatedly shown that no new therapeutic approach that was successfully tested in preclinical models was effective in clinical practice (39). Our model replicates many of the biological features intrinsic to human septic shock and integration of standard day-to-day care resuscitative measures makes it an appealing sepsis model in translational research. The International Society for Stem Cell Research has recently released guidelines for clinical translation stating strongly that large-animal models should be used for stem cell research and that any clinical trial should be preceded by compelling preclinical evidence obtained from these models (40). The clinical application of MSCs that we are witnessing in the field of orthopedics or neurology, and which is based on minimal evidence of benefit and safety, represents a path that critical care medicine should avoid (1). Generally homogenous and encouraging results attained by current preclinical testing cannot be considered as sufficient arguments for launching clinical trials, in part, due to a considerable risk that the effect of MSCs is overstated given that a number of studies with negative results have not been published.

## Conclusion

In this large, clinically relevant animal peritonitis-induced sepsis model, MSCs were not capable of reversing any of the sepsis-induced disturbances in multiple biological, organ, and cellular systems. Collectively, our study cautions against the use of MSCs in a complex disease such as sepsis, as our understanding of the role MSCs play in it is still incomplete and unknown factors may influence the outcome.

## Data Availability Statement

The datasets generated for this study are available on request to the corresponding author.

## Ethics Statement

The animal study was reviewed and approved by the Committee for Experiments on Animals of the Faculty of Medicine, Charles University, in Pilsen and by the Ministry of Education, Youth and Sports of the Czech Republic (protocol no. MSMT-20064/2015-3).

## Author Contributions

JH, LN, VM, VT, and JB performed animal experiments. MM and MS conceived and designed the study. MM, MS, LV, and JH performed data analysis. MM and JH drafted the manuscript. LV and MD performed MSC processing and flow cytometry measurements. JK and MK performed mitochondrial function measurement and data analysis. MS and DJ performed cardiac myocytes measurements and data analysis. All authors contributed to data interpretation, editing of the manuscript, and read and approved the final manuscript.

### Conflict of Interest

The authors declare that the research was conducted in the absence of any commercial or financial relationships that could be construed as a potential conflict of interest.

## Abbreviations

ALT: Alanine aminotransferase
AST: Aspartate aminotransferase
CVP: Central venous pressure
DO_2_: Systemic oxygen delivery
EDTA: Ethylenediaminetetraacetic acid
hsTnT: High-sensitivity troponin T
IL-6: Interleukin 6
IL-8: Interleukin 8
IL-10: Interleukin 10
MAP: Mean arterial pressure
MSCs: Mesenchymal stem cells
PAOP: Pulmonary artery occlusion pressure
PaO_2_/FiO_2_: Oxygenation index
PBS: Phosphate-buffered saline
SOFA: Sepsis-related organ failure assessment
TNF-α: Tumor necrosis factor α
VO_2_: Systemic oxygen uptake.
