# Supplementary material for: Evaluation of Mesenchymal Stem Cell Therapy for Sepsis: A Randomized Controlled Porcine Study
Source: Front Immunol. 2020 Feb 7;11:126. doi: 10.3389/fimmu.2020.00126 (PMC7019005; doi:10.3389/fimmu.2020.00126)
Supplement: Supplementary file 1 [file Data_Sheet_1.pdf]

Supplemental digital material

Evaluation of mesenchymal stem cell therapy for sepsis: a randomized controlled porcine study

Materials and methods

2.5. Monitoring, sampling and measurements

Pigs were monitored by Mindray BeneVision T9 (Mindray, Shenzen, China). Systemic and regional hemodynamic parameters were measured by using standardized devices – arterial cathether (Arrow/Teleflex Europe, Dublin, Ireland) and pulmonary artery cathether (BBraun, Bethlehem, Pennsylvania, USA). Direct measurement of ECG-heart rate (HR), mean arterial pressure (MAP), central venous pressure (CVP), mean pulmonary artery pressure (MPAP) and pulmonary artery wedge pressure (PAWP) were performed. Cardiac output (CO) was measured by thermodilution method. Delivery of oxygen (DO_2) and oxygen consumption (VO_2) were calculated:

$$\mathbf{DO_2 = CO/kg \times (1{,}34 \times Hb \times SaO_2 + 0{,}003 \times PaO_2) / 100}$$

$$\mathbf{VO_2 = CO/kg \times (1{,}34 \times Hb \times (SaO_2 – SvO_2) + 0{,}003 \times (PaO_2 – PvO_2)) / 100}$$

Based on measurements of systemic hemodynamic parameters we calculated systemic vascular resistance (SVR):

$$\mathbf{SVR = 80 \times (MAP – CVP) / CO}$$

Renal artery blood flow (RBF) was measured by using the Doppler ultrasound flow probe (Transonic Systems, Ithaca, NY, USA). Due to cardiac output variability it was modified and presented as RBF to CO ratio (RBF/CO).

Supplemental material - Table antibodies MSC: Antibodies used for MSCs phenotype control.

ANTIBODIES	MANUFACTURER	CAT. NUMBER	CLONE	REACTIVITY	ISOTYPE
CD90-APC	Biolegend	1A-625-T025	5E10	pig, horse, human	IgG1
CD73-PE	Biolegend	1P-675-T025	AD2	human, pig	IgG1
CD44-BV421	Biolegend	103040	IM7	mouse, human, pig	IgG2b
CD45 FITC	Abdserotec	MCA1222F	K252.1E4	pig	IgG1

Supplemental material - Table antibodies: Antibodies used for leukocytes subpopulation flow cytometry analysis.

LEUKO subpopulations	ANTIBODIES	MANUFACTURER	CAT. NUMBER	CLONE	REACTIVITY	ISOTYPE
$CD14^{pos}CD16^{pos}$ monocytes	CD14 APC	Milteney Biotech	130-091-243	REA599	pig, sheep, goat	IgG1
	CD16 PE	AbdSerotec	MCA1971PE	G7	pig	IgG1
Th and Tc lymphocytes	CD3ε PE-Cy7	Becton Dickinson	561477	BB23-8E6-8C8	pig	IgG2a
	CD8α Pe-Cy5	Abcam	ab25536	76-2-11	pig	IgG2a
	CD4α Alexa Fluor 647	Becton Dickinson	561472	74-12-4	pig	IgG2a
Treg	CD4α Alexa Fluor 647	Becton Dickinson	561472	74-12-4	pig	IgG2a
	FoxP3 PE-Cy7	eBiosciences	25-5773-82	FJK-16s	pig, rat, dog	IgG2a
	CD25 unconj. + IgG1 BV421	AbdSerotec	MCA1736GA	K231.3B2	pig	IgG1
	secondary Ab IgG1 BV421	eBioscienc	562580	A85-1	mouse	IgG1

Supplemental material - Table 1. Blood count and inflammatory parameters in sham-operated animals with and without MSC administration. The (*) stands for statistical significance in time. The (#) stands for statistical significance between the groups.

Parameter	Timepoint	CONTROL	MSC-CONTROL
**Leukocytes (1×10^9/L)**	**Baseline**	19 (17-21)	20 (17-22)
	**+12 H**	15 (13-16)*	15 (14-17)*
	**+18 H**	8 (6-13)*	12 (10-15)*
	**+24 H**	11 (8-12)*	13 (8-16)*
**Erythrocytes (1×10^9/L)**	**Baseline**	5,1 (4,9-5,5)	5,2 (5,0-6,5)
	**+12 H**	4,7 (4,3-5,1)*	4,6 (4,5-5,0)*
	**+18 H**	4,5 (4,0-4,7)*	4,3 (4,2-4,7)*
	**+24 H**	4,6 (4,1-5,4)*	4,4 (3,9-5,8)*
**Trombocytes (1×10^9/L)**	**Baseline**	285 (226–315)	260 (231–357)
	**+12 H**	227 (204–302)	200 (191–236)*
	**+18 H**	236 (213–271)	201 (164–220)*
	**+24 H**	241 (216–252)	194 (190–226)*
**Lymphocytes (%)**	**Baseline**	35 (19-55)	29 (15-50)
	**+12 H**	60 (48-72)	61 (58-64)*
	**+18 H**	53 (45-62)	62 (44-70)*
	**+24 H**	46 (45-55)	46 (40-58)*
**Monocytes (%)**	**Baseline**	0,5 (0,4-0,6)	0,4 (0,4-1,0)
	**+12 H**	0,4 (0,3-0,5)#	1,0 (0,6-1,6)#
	**+18 H**	0,6 (0,2-0,8)	0,4 (0,3-0,5)
	**+24 H**	0,6 (0,4-1,5)	2,1 (0,7-3,9)
**Neutrophils (%)**	**Baseline**	Not available	Not available
	**+12 H**	Not available	Not available
	**+18 H**	Not available	Not available
	**+24 H**	Not available	Not available
**Eosinophils (%)**	**Baseline**	0,1 (0 – 0,3)	0,1 (0 – 0,3)
	**+12 H**	2,8 (1,8-4,4)*	2,7 (0,3-4,3)*
	**+18 H**	4,5 (1,5-8,6)*	5,2 (3,5-6,9)*
	**+24 H**	3,8 (2,1-7,7)*	3,9 (3,3-8,8)*
**Basophils (%)**	**Baseline**	0	0
	**+12 H**	0 (0-0,5)	0
	**+18 H**	0 (0-1,6)	0 (0-1,2)
	**+24 H**	1,2 (0-2,8)	2,1 (0-6,2)
**C-reactive protein (mg/L)**	**Baseline**	0,4 (0,3-0,7)	0,3 (0,1-0,5)
	**+12 H**	1,2 (1,0-1,5)	1,1 (0,6-1,4)
	**+18 H**	1,4 (1,1-1,9)	1,0 (0,7-1,7)
	**+24 H**	1,5 (0,9-1,8)	1,0 (0,9-1,9)
**IL-8 (ng/L)**	**Baseline**	31,9 (4,9-55,1)	37,4 (32,0-70,0)
	**+12 H**	39,4 (28,1-48,9)	36,1 (28,4-49,5)
	**+18 H**	57,5 (47,7-119,9)	40,4 (36,2-45,2)
	**+24 H**	25,6 (21,9-44,2)	25,4 (22,9-54,5

Supplemental material - Table 2. Blood count and inflammatory parameters in septic animals with and without MSC administration. The (*) stands for statistical significance in time. The (#) stands for statistical significance between the groups.

Parameter	Timepoint	SEPSIS	MSC-SEPSIS
**Leukocytes (1×10^9/L)**	**Baseline**	20 (13-26)	20 (15-22)
	**+12 H**	7 (4-11)*	9 (8-15)*
	**+18 H**	5 (4-6)*	8 (5-9)*
	**+24 H**	7 (5-9)*	7 (3-9)*
**Erythrocytes (1×10^9/L)**	**Baseline**	5,7 (5,3-5,9)	5,8 (4,9-6,4)
	**+12 H**	6,2 (5,6-6,6)	6,5 (6,3-6,9)*
	**+18 H**	6,0 (5,6-7,6)	6,8 (5,5-7,2)*
	**+24 H**	6,9 (6,5-7,2)	7,3 (6,9-7,7)*
**Trombocytes (1×10^9/L)**	**Baseline**	284 (254-349)	310 (190-355)
	**+12 H**	194 (177-255)*	206 (127-256)*
	**+18 H**	139 (132-210)*	181 (104-183)*
	**+24 H**	120 (93-140)*	112 (69-151)*
**Lymphocytes (%)**	**Baseline**	39 (25-58)	25 (14-58)
	**+12 H**	43 (39-52)	44 (40-48)
	**+18 H**	52 (44-57)	51 (34-54)
	**+24 H**	27 (10-45)	52 (31-60)
**Monocytes (%)**	**Baseline**	1,1 (0,8-2,2)	0,5 (0,3-0,6)#
	**+12 H**	3,4 (0,3-4,4)	1,4 (0,5-3,7)
	**+18 H**	3,3 (1,9-3,5)	2,3 (1,3-5,1)
	**+24 H**	3,8 (2,4-4,8)	2,1 (1,4-2,9)
**Neutrophils (%)**	**Baseline**	Not available	Not available
	**+12 H**	Not available	Not available
	**+18 H**	Not available	Not available
	**+24 H**	Not available	Not available
**Eosinophils (%)**	**Baseline**	0,1 (0-0,2)	0
	**+12 H**	0,6 (0,5-0,8)	0,6 (0,4-1,1)*
	**+18 H**	3,5 (3,1-3,6)*	7,0 (1,1-7,6)*
	**+24 H**	5,4 (4,0-11,5)*	5,4 (3,5-7,6)*
**Basophils (%)**	**Baseline**	0	0
	**+12 H**	0,8 (0,3-1,2)*	0,5 (0,2-0,7)
	**+18 H**	0,3 (0,2-0,5)	0,5 (0-0,6)
	**+24 H**	0,4 (0,3-0,4)	0,5 (0,2-0,6)
**C-reactive protein (mg/L)**	**Baseline**	0,3 (0,2-0,4)	0,3 (0,2-0,6)
	**+12 H**	0,9 (0,5-1,1)*	1,0 (0,8-1,1)*
	**+18 H**	1,0 (0,6-1,6)*	1,1 (0,9-1,5)*
	**+24 H**	1,0 (0,6-1,4)*	1,1 (0,8-1,5)*
**IL-8 (ng/L)**	**Baseline**	38,9 (30,7-72,5)	34,7 (30,4-149,1)
	**+12 H**	39,5 (24,9-83,7)*	35,6 (29,4-80,4)*
	**+18 H**	55,5 (37,9-118,4)*	48,0 (33,9-76,9)*
	**+24 H**	33,7 (28,0-183,0)*	45,5 (36,8-50,1)*
**8-isoprostane (µg/L)**	**Baseline**	59 (23-146)	17 (4-52)
	**+12 H**	653 (367-1351)*	364 (178-466)*
	**+18 H**	922 (468-1644)*	639 (241-1673)*
	**+24 H**	1409 (574-2045)*	1576 (1003-2269)*
